# Histone deacetylases and their role in motor neuron degeneration

**DOI:** 10.3389/fncel.2013.00243

**Published:** 2013-12-05

**Authors:** Rafael Lazo-Gómez, Uri N. Ramírez-Jarquín, Luis B. Tovar-y-Romo, Ricardo Tapia

**Affiliations:** División de Neurociencias, Instituto de Fisiología Celular, Universidad Nacional Autónoma de México, D. F.México

**Keywords:** amyotrophic lateral sclerosis, neurodegeneration, neuroprotection, transcription dysregulation, histone deacetylases, sirtuins

## Abstract

Amyotrophic lateral sclerosis (ALS) is a fatal neurodegenerative disease, characterized by the progressive loss of motor neurons. The cause of this selective neuronal death is unknown, but transcriptional dysregulation is recently emerging as an important factor. The physical substrate for the regulation of the transcriptional process is chromatin, a complex assembly of histones and DNA. Histones are subject to several post-translational modifications, like acetylation, that are a component of the transcriptional regulation process. Histone acetylation and deacetylation is performed by a group of enzymes (histone acetyltransferases (HATs) and deacetylases, respectively) whose modulation can alter the transcriptional state of many regions of the genome, and thus may be an important target in diseases that share this pathogenic process, as is the case for ALS. This review will discuss the present evidence of transcriptional dysregulation in ALS, the role of histone deacetylases (HDACs) in disease pathogenesis, and the novel pharmacologic strategies that are being comprehensively studied to prevent motor neuron death, with focus on sirtuins (SIRT) and their effectors.

## Introduction

Amyotrophic lateral sclerosis (ALS) is a fatal neurodegenerative disease characterized by the death of upper and lower motor neurons. There are two type of ALS based on the presence of an identified mutation as a cause of the disease. The familial type (fALS) is most commonly caused by mutations in the superoxide dismutase type 1 (SOD1) gene, for which there is a widely studied transgenic mouse model; however fALS represents only about 10% of cases. The most common type of ALS is called sporadic (sALS), but because there are no identified etiologic factors, validated experimental models lack for this type of the disease. Nevertheless, disease progression and pathologic characteristics in both, fALS and sALS, are similar. Also, in both types of the disease, motor neuron degeneration has been associated with the same mechanisms, such as glutamate-mediated excitotoxicity, inflammatory events, axonal transport deficits, oxidative stress and mitochondrial dysfunction (for recent reviews see Corona et al., 2007; Santa-Cruz et al., 2012).

Besides these factors, a novel mechanism that may be involved in motor neuron death and other neurodegenerative diseases is transcriptional dysfunction, which consists of aberrations of the molecular machinery that regulates gene expression, especially at the transcriptional levels through the manipulation of epigenetic marks (Robberecht and Philips, 2013). These marks are covalent modifications of the chromatin components, DNA and histone proteins, carried out by several key enzymes that finely modulate the status of these marks. Among these marks, histone acetylation has been characterized as an important mechanism in the regulation of the opening of chromatin configuration leading to increased transcription (Kouzarides, 2007). The status of histone acetylation strongly depends on the activity of histone deacetylases (HDACs), a widely conserved family of enzymes that catalyze the removal of acetyl groups from histones and from other proteins. This family, which includes sirtuins (SIRT), has been shown to be important for several cellular processes, such as cell death and stress responses, which makes them attractive for the study of their pathogenic role and as potential therapeutic targets (Haigis and Sinclair, 2010).

This paper will review, first the evidence of transcriptional dysregulation in ALS, then the role of the different members of the HDACs family in disease pathogeny and as therapeutic candidates, and finally the role of SIRT and their effectors in ALS.

## Relevance of histone acetylation in gene expression

Protein acetylation is an important postranslational modification that regulates numerous cellular functions, such as microtubule dynamics and intracellular transport (Hubbert et al., 2002), development (Bhaumik et al., 2007), metabolism (Cantó et al., 2009), and transcriptional regulation through chromatin remodeling (Kouzarides, 2007).

In eukaryotic cells, including neurons, the genome is organized through the association of DNA with protein complexes, an assembly called chromatin. The most abundant proteins are histones, and these proteins assemble themselves into octamers, composed of two copies of the histones types H2A, H2B, H3 and H4. Around the octamer, ∼147 bp of DNA is wrapped, forming the basic unit of chromatin, the nucleosome. Nucleosomes further organize themselves in increasingly complex structures, all of which are subject to modulation, by changing the accessibility of the regulatory regions of DNA (such as promoters, enhancers, silencers or insulators) to the transcriptional machinery, ultimately affecting gene expression (Zhou et al., 2011). This regulation can be accomplished through covalent modifications of the chromatin components: in the DNA it occurs through cytosine methylation and hydroxymethylation, and in the histones through methylation, ubiquitinylation, sumoylation, phosphorylation and acetylation of selected aminoacid residues (Kouzarides, 2007). In general terms, gene expression is directly proportional the level of histone acetylation; because of this, the regulation of this postranslational modification is essential for gene homeostasis. Such regulation depends on two groups of enzymes: histone acetyltransferases (HATs) and HDACs, and drastic alterations to this control of gene expression can be deleterious, as has been proven in various tumor cell models (Frew et al., 2009). Therefore, the upregulation of the expression of a given gene can be accomplished by stimulating the activity of HATs or by inhibiting the activity of HDACs (Kazantsev and Thompson, 2008). Currently, research on HATs and on their possible protective effects on neurodegenerative processes is scarce and no specific activators are known, whereas information on HDACs has become the subject of many studies (Selvi et al., 2010). Although histones are not the only target of these enzymes, and in some instances they are not a target at all, the name HDACs is preserved for historical reasons. They constitute a family integrated in a complex network of intracellular signaling participating through acetylation/deacetylation reactions, with numerous protein substrates and multiple physiological consequences depending on the protein involved (de Ruijter et al., 2003; Kazantsev and Thompson, 2008).

## Evidence of transcriptional dysregulation in ALS

There is evidence that transcriptional dysregulation could contribute to, and possibly be the cause of, some neurodegenerative disorders. This was first proposed for Huntington’s disease (Cha, 2000) and relevant findings have been encountered in experimental in vitro and in vivo models of Alzheimer’s (Robakis, 2003) and Parkinson’s diseases (Yacoubian et al., 2008). In the case of ALS, there is also evidence of transcriptional dysregulation and alterations in the transcriptome in both the sporadic (Figueroa-Romero et al., 2012) and the familial (Kirby et al., 2005) types of the disease. Although the mechanisms and causal relationships have yet to be elucidated, it has been proposed that neuronal protein inclusions, such as those formed by mutated SOD1 or TDP43 (43-kDa transactivator response region (TAR) DNA-binding domain protein) which have been found in ALS and in frontotemporal dementia, could cause their toxicity by acting as surface attractants that sequester vital components of the transcriptional machinery, as was first proved for huntingtin (Cha, 2000). For example, in a screening study to identify targets that disrupt SOD1 aggresome in cultured cells, HDAC inhibition was shown to prevent aggresome formation (Corcoran et al., 2004).

Transcription might also be altered indirectly by modifications of other regulatory components of the transcriptional process, such as HDAC activity. For example, in a *Drosophila* model of polyglutamine neurodegeneration, it was shown that the upregulation of HDACs can ameliorate neuronal death due to a selective transcriptional repression of the CGG repeat-containing gene (Todd et al., 2010). Also, HDAC inhibition is protective for cultured motor neurons against excitotoxicity, a mechanism known to be involved in ALS pathophysiology, due to their ability to modulate gene expression (Kanai et al., 2004). Hence, although the role of transcriptional dysfunction in neurodegenerative diseases is still under study, there is increasing evidence for a role of HDACs in the neurodegenerative processes through the modulation of transcriptional machinery.

## Histone deacetylases: role of classes I and II in motor neuron degeneration

Based on structural, localization and functional criteria, HDAC superfamily is composed of five classes. First, in the next paragraphs, we will focus on the “typical” HDACs (11 enzymes, classified in classes I, IIa, IIb and IV), their distribution and the evidence of their role in ALS. The role of SIRT (seven enzymes that constitute class III or “atypical” HDACs) will be addressed later.

Class I and II HDACs are Zn^2+^-dependent enzymes. Class I include HDAC1, HDAC2, HDAC3 and HDAC8, which are localized in the nucleus and ubiquitously expressed in mammalian tissues (except HDAC8, which is muscle-specific). Broadly speaking, these enzymes are involved in the regulation of gene-specific transcription through the formation of stable transcriptional complexes (de Ruijter et al., 2003). Of these, HDAC2 and HDAC3 seem to have a more important role in the physiology of the central nervous system. Broide et al. (2007) described the distribution of 11 HDACs mRNAs in 50 screened areas of the rat brain, using high-resolution in situ hybridization; HDAC2 and HDAC3 were widely expressed in all areas, especially those of the limbic system (amygdala, piriform cortex, olfactory bulb and hippocampus) and in the granule cell layer of cerebellum, and at the cellular level were found in neurons and oligodendrocytes. In a recent report, a similar distribution of HDAC2 was found in the mouse brain, as well as a moderate expression in primary and secondary motor cortices, and Rexed’s laminae 4–9 of the cervical spinal cord (where motor neurons reside). Again, HDAC2 was noted only in neurons and oligodendrocytes, within the nucleus (Yao et al., 2013). Due to their ubiquitous expression in brain and spinal cord, these HDACs have also been implicated in ALS. Janssen et al. (2010) found in ALS patients that HDAC2 expression was upregulated in motor cortex (in layers III–V, where upper motor neuron are located) and in spinal cord grey matter, particularly in the nuclei of motor neurons; they interpreted this result as a protective role of HDAC2 in ALS pathogenesis, although the mechanisms of this effect were not detailed. Regarding this issue, in an in vitro study Kernochan et al. (2005) reported that HDAC inhibition increased promoter activity of the survival motor neuron 2 gene, and this was associated with HDAC2 levels.

Class IIa –HDACs 4, 5, 7 and 9– shuffle between the nucleus and the cytoplasm, and their substrates have not been defined; they have histone deacetylase activity only by interacting with HDAC3 (de Ruijter et al., 2003). HDAC4 and 5 expressions were high in all screened areas, and their locations mimic that of class I HDACs (Broide et al., 2007).

Class IIb is composed of HDAC6 and HDAC10. Again, HDAC10 substrates have not been defined, but it is known that it associates with HDAC3 (de Ruijter et al., 2003). HDAC6 is an unusual enzyme, in the sense that it has two catalytic domains and functions in the cytoplasm where it deacetylates *α*-tubulin and alters microtubule stability (Hubbert et al., 2002). Furthermore, inhibition of this enzyme stimulates autophagy and the proteasome system in a *Drosophila* model of spinal muscular atrophy (a neurodegenerative disease of spinal motor neurons) resulting in increased survival (Pandey et al., 2007). In the rat brain, its expression is low (Broide et al., 2007) and no changes were noted in ALS patients (Janssen et al., 2010). Nevertheless, evidence of the role of HDAC6 in ALS is emerging. In a recent work it was found that TDP43 and fused in sarcoma/translated in liposarcoma (FUS/TLS), which are proteins that regulate RNA processing and have been found to be mutated in some cases of ALS, interact with each other forming a ribonucleoprotein complex that regulates the expression of HDAC6 through its mRNA stability (Kim et al., 2010).

Class IV HDAC only member, HDAC11, is localized in the cell nucleus and is structurally different from the other classes (Kazantsev and Thompson, 2008). Although its functions are poorly understood, recent reports indicate that this HDAC is abundantly and almost exclusively expressed in the mammalian nervous system, within oligodendrocytes (Liu et al., 2008), and also plays an important role in the maturation of this cell type (Liu et al., 2009b). In other study in the rat brain, HDAC11 expression was found to be ubiquitously distributed, in both neurons and oligodendrocytes; in fact, this HDAC displayed the highest levels of expression of all HDACs (Broide et al., 2007). In nervous tissue of ALS patients, including the ventral horn of the spinal cord, the nucleus and the cytoplasm of motor neurons showed decreased HDAC11 mRNA levels (Janssen et al., 2010).

## Protective effects of HDAC inhibition

Chen et al. (2012) addressed the role of class I and II HDACs in an experimental model of acute stroke in rodents. There was progressive decline of mRNA levels of HDACs 1, 2, 5 and 9 in cerebral cortex, while HDACs 3, 6 and 11 mRNA levels were transiently increased. In addition, in an in vitro model of glucose-oxygen deprivation, they reported that selective inhibition of HDAC3 and HDAC6 promoted neuronal survival.

Currently there is an ample battery of small-molecule HDACs inhibitors, that were initially developed to halt cell proliferation in cancer experimental models; HDACs inhibitors are classified as hydroxamate-based (vorinostat, valproic acid (VPA), sodium butyrate, trichostatin A, 4-phenyl butyrate, MC1568 –class II selective–, and tubucin –HDAC6 specific–), and benzamide-based (MS275, compound 106). Their potencies and selectivities vary, but most of them can be considered pan-HDACs inhibitors (Kazantsev and Thompson, 2008).

Several of these compounds have been studied in the transgenic SOD1 mouse model. Trichostatin A induced a modest improvement in motor function and survival as well as protection against motor neuron death, axonal degeneration, muscle atrophy and neuromuscular junction denervation; these effects were attributed to reduced gliosis and upregulation of the glutamate transporter (GLT-1) in the spinal cord (Yoo and Ko, 2011).

VPA is a drug currently in clinical use for other neurological disorders (such as epilepsy and bipolar disorder) due to its numerous mechanisms of action, including HDAC inhibition (Monti et al., 2009). VPA was tested in vivo in the transgenic ALS SOD1 mice, where it did not improve survival or motor performance, but it did improve the acetylation status in the spinal cord through the restoration of the cAMP response element binding protein (CREB) levels in motor neurons and slightly prevented motor neuron death (Rouaux et al., 2007). VPA has already been tested in ALS patients in one clinical trial, showing no benefits in survival or in disease progression in doses commonly used to treat epilepsy (Piepers et al., 2009).

Sodium phenylbutyrate (SPB), another pan-HDAC inhibitor, was shown to extend survival and motor performance in the transgenic ALS SOD1 animal model, and these effects were attributed to an upregulation in the expression of nuclear factor *κ*B (NF-*κ*B), the active form of the inhibitory subunit of NF-*κ*B (i-*κ*B) and of beta cell lymphoma 2 (bcl-2) proteins, all involved in survival and stress responses (Ryu et al., 2005). SPB efficacy has been tested also in combinations with other agents with different mechanisms of action, in the same experimental model. For example, the combination of SPB with riluzole potentiated the beneficial effects of SPB on survival and motor performance, although the authors did not address the possible mechanisms (Del Signore et al., 2009). SPB has also been tested in combination with an antioxidant agent (AEOL 10150), where it was found that this combination extended survival more than either treatment alone (Petri et al., 2006). Finally, it is worth to emphasize that SPB has already been tested in a phase 2 clinical trial, where its effects on histone acetylation status, safety and tolerability were addressed; no toxic effects were noted, and significant increases were observed in the blood histone acetylation status, but the therapeutic efficacy of such treatment was not studied (Cudkowicz et al., 2009).

## Histone deacetylases: role of sirtuins in motor neuron degeneration

Class III HDACs, also called SIRT, are the most divergent class from the rest of the family. This class includes seven members (SIRT1 to SIRT7) with various cellular localizations and preferred enzymatic targets, but what makes unique this class is the dependence of nicotine adenine dinucleotide (NAD^+^) to perform their catalytic deacetylation and mono-ADP-ribosyl transferase activities (Michan and Sinclair, 2007). SIRT1, SIRT2, SIRT3 and SIRT5 have predominant histone deacetylase activity (Haigis and Sinclair, 2010). SIRT1, SIRT6 and SIRT7 are located in the nucleus. SIRT1 has a preference for euchromatin and can be shuttled to the cytoplasm, depending on the cell type and the developmental stage (Tanno et al., 2007). SIRT6 tends to associate with heterochromatin and SIRT7 is located in the nucleolus. SIRT2 resides mostly in the cytoplasm, having an important role in regulation cytoskeletal dynamics. SIRT3, SIRT4 and SIRT5 are mitochondrial (Michan and Sinclair, 2007).

SIRT are named after the silent information regulator 2 (sir2) gene in yeast. When overexpressed, the product of this gene extended the lifespan of budding yeast by repressing genomic instability (Kaeberlein et al., 1999). After that discovery, it was learned that SIR2-like genes or SIRT are found in most organisms, where they regulate basic vital functions. In mammals, sirtuin activation or overexpression do not extend lifespan, but appear to be involved in the benefits of calorie restriction, are responsive to environmental stimuli (e.g., daylight and cell stress), and play important roles in numerous human diseases, such as cancer, diabetes, cardiovascular disease and neurodegeneration (Haigis and Sinclair, 2010).

The catalytic deacetylase activity of SIRT is performed on histones and on other proteins, and this ability place SIRT in a privileged position to exert their actions on the genome through direct and indirect pathways. Among the non-histone protein substrates, the peroxisome proliferator-activated receptor gamma coactivator 1 alpha (PGC1-*α*; Cantó and Auwerx, 2009) and the forkhead box O3a (FoxO3a) transcription factor (Zhao et al., 2011b) are two of the most studied, and their relation to ALS will be discussed below. Also, SIRT play an important role as cellular energy sensors (Smith et al., 2000), due to their dependence on NAD^+^ as a substrate (Imai et al., 2000); in humans, SIRT1-3 and 5 have shown in vitro NAD^+^-dependent deacetylating activity (Smith et al., 2000). Therefore, SIRT provide a connection between cellular energy states and transcriptional control (Li, 2013).

SIRT have a widespread distribution in the mammalian tissues, including the nervous system. In the mouse, rat and human CNS, SIRT1 mRNA localization was found to be ubiquitous and prominent in various tissues (such as all regions of spinal cord, hippocampus, basal ganglia, brain stem and cerebellum); SIRT1 expression is confined to the neuronal nucleus in parvalbumin (GABAergic) and tyrosine hydroxylase (dopaminergic) neurons (Zakhary et al., 2010). SIRT1 expression was increased in cerebral cortex (especially in the pyramidal cell layer), hippocampus, thalamus and spinal cord in symptomatic SOD1 G93A mice, but spinal motor neurons were not studied in detail; these changes were interpreted as a stress response to the neurotoxic form of SOD1 (Lee et al., 2012).

## Protective effects of sirtuins activation

In contrast to other HDACs, the activity of SIRT in the CNS results in neuroprotection. Among the agents with protective action are the polyphenols (vegetal compounds that serve hormonal and protective function in these organisms), particularly resveratrol, which is present in grapes and in red wine, and SRT1720, a synthetic compound derived from resveratrol. These compounds activate selectively both SIRT1 and SIRT2, whereas activators of the remaining SIRT have not yet been described (Kazantsev and Thompson, 2008).

There is a paucity of studies that address SIRT role in ALS pathology. In primary cortical cultures of transgenic SOD1 G93A mice, resveratrol protected against neuron death, and this protection was due to the activation of SIRT1 (Kim et al., 2007). In agreement with these results, in another in vitro study of SOD1 G93A murine motor neurons, it was found that SIRT1 expression was downregulated, and resveratrol prevented neuronal death (Wang et al., 2011). However, the neuroprotection exerted by resveratrol may be due to the stimulation of the activities of other enzymes such as AMP protein kinase (Dasgupta and Milbrandt, 2007). In our laboratory we have recently found that the administration of resveratrol directly in the lumbar spinal cord in rats delays the progress of motor deficits induced by chronic AMPA (*α*-amino-3-hydroxy-5-methyl-4-isoxazol-propionate) infusion in the lumbar spinal cord (Lazo-Gómez and Tapia, in preparation, an in vivo model of excitotoxic motor neuron death (Tovar-y-Romo et al., 2007).

The downstream effectors of SIRT that confer protection in neurodegenerative settings are beginning to be elucidated. For example, in a transgenic mouse model of Huntington’s disease, SIRT1 overexpression exerted its beneficial effects through the deacetylation of FoxO3a and p53 (Jiang et al., 2012). This relation has not been explicitly addressed in ALS experimental models, but there is evidence of the role of SIRT1 effectors in motor neuron death. FoxO3a is a transcriptional factor involved in determining cell fate (survival or apoptosis) in stressful situations, such as starvation and oxidative stress. It has been shown that SIRT1 modulates FoxO3a activities through the deacetylation of specific lysine residues, resulting in activation or inhibition, and this depends on the deacetylated lysine position or on FoxO3a cellular location (nucleus or cytoplasm) (Eijkelenboom and Burgering, 2013). In an in vitro study of motor neuron death induced by several insults (excitotoxicity, the overexpression of mutant SOD1, of mutant p150^glued^ or of poly-glutamin expanded androgen receptor), targeted expression of FoxO3a to the nucleus (where it can modulate the transcription of its target genes) by genetic or pharmacologic means prevented neuron death (Mojsilovic-Petrovic et al., 2009). This is in contrast with other in vitro studies, where motor neuron cultures deprived of trophic support were found to overexpress Fas ligand (FasL), due to FoxO3a translocation to the nucleus, thus resulting in cell death (Barthélémy et al., 2004). The reason for this discrepancy is not clear, but it could be due to differences in the death process triggered by the various insults used, which might be regulated in different ways by FoxO3a.

Another well studied SIRT1 effector is PGC1-*α*, a transcriptional coactivator involved in the modulation of the responses necessary to overcome cellular energetic deficiencies, such as the stimulation of mitochondrial biogenesis and the respiratory rate, and the increase of the uptake and metabolism of energy substrates. SIRT1 physically interacts with, deacetylates and activates PGC1-*α* (Cantó and Auwerx, 2009). It has been shown that PGC1-*α* plays a role in ALS pathology, because decreased mRNA and protein levels of this coactivator were found in both transgenic mice and in patients with the sporadic form of the disease, in muscle and in spinal cord tissues (Thau et al., 2012). Moreover, PGC1-*α* may play a therapeutic role, as shown by two studies in the transgenic mouse model. Zhao et al. (2011a) used a targeted overexpression of PGC1-*α* in neurons of SOD1 transgenic mice, which modestly increased survival and significantly improved motor performance; these changes were due to the restoration of mitochondrial activities. This is in contrast to the findings of other study in a double transgenic mouse overexpressing PGC1-*α* and mutant SOD1 in all tissues, showing that survival was not increased albeit motor performance improved and motor neuron loss was ameliorated. An augmentation of the excitatory amino acid transporter protein 2 (EAAT2) in astrocytes was noted in the double transgenic, suggesting that PGC1-*α* could exert its beneficial effects through other mechanisms (Liang et al., 2011). Regarding other sirtuin isoforms, such as SIRT3, a mitochondrial sirtuin, Song et al. (2013) showed, in primary spinal motor neuronal cell cultures of transgenic SOD1 G93A mice, that the overexpression of SIRT3 and of PGC1-*α* protected against mitochondrial fragmentation and neuronal cell death, although the authors did not demonstrate the relation between SIRT3 and PGC1-*α*.

These findings indicate that sirtuin activation protects against motor neuron degeneration, although some findings in different experimental models suggest that activators may protect through other mechanisms (Tang, 2010) or even that sirtuin inhibition may be protective. For example, in a model of brain ischemia sirtuin inhibition through nicotinamide administration conferred neuroprotection by preserving NAD^+^ cellular levels (Liu et al., 2009a).

## Conclusion

The opportunities for a really effective therapy for ALS are scarce, and therefore HDAC inhibition and sirtuin activation merit further investigation. Studies on the modification of the activity of these enzymes in in vitro and in vivo experimental models of neurodegeneration, including ALS, have given valuable information suggesting potential therapeutic opportunities for this disease. Nevertheless, the mechanisms underlying such effects are poorly understood and their clarification is necessary for designing more specific and potent treatment strategies. Accumulated evidence on sirtuin activation has provided valuable information about the molecular pathways that could be relevant to halt motor neuron degeneration, such as those related to cellular energetic state and PGC1-*α*. Not all types of HDACs have been evaluated in ALS models and the effects, toxicity, dosage, timing, and mode of administration of specific drugs that inhibit HDAC or activate SIRT have not been established. Of note is the fact that the most widely used model of ALS in which these strategies have been studied is the transgenic rodent with mutations in the human SOD1 gene, but this represent only about 2% of all ALS cases. Novel experimental models of motor neuron degeneration in vivo are clearly needed before safely moving these drugs to clinical trials for a disorder that has proved recalcitrant to all, past and current, therapeutic procedures.

## Conflict of interest statement

The authors declare that the research was conducted in the absence of any commercial or financial relationships that could be construed as a potential conflict of interest.

## References

[B1] Barthélémy C. Henderson C. E. Pettmann B. (2004). Foxo3a induces motoneuron death through the Fas pathway in cooperation with JNK. BMC Neurosci. 5:48 10.1186/1471-2202-5-48 15569384 PMC538283

[B2] Bhaumik S. R. Smith E. Shilatifard A. (2007). Covalent modifications of histones during development and disease pathogenesis. Nat. Struct. Mol. Biol. 14, 1008–1016 10.1038/nsmb1337 17984963

[B3] Broide R. S. Redwine J. M. Aftahi N. Young W. Bloom F. E. Winrow C. J. (2007). Distribution of histone deacetylases 1–11 in the rat brain. J. Mol. Neurosci. 31, 47–58 10.1007/bf02686117 17416969

[B4] Cantó C. Auwerx J. (2009). PGC-1alpha, SIRT1 and AMPK, an energy sensing network that controls energy expenditure. Curr. Opin. Lipidol. 20, 98–105 10.1097/mol.0b013e328328d0a4 19276888 PMC3627054

[B5] Cantó C. Gerhart-Hines Z. Feige J. N. Lagouge M. Noriega L. Milne J. C. (2009). AMPK regulates energy expenditure by modulating NAD+ metabolism and SIRT1 activity. Nature 458, 1056–1060 10.1038/nature07813 19262508 PMC3616311

[B6] Cha J. H. (2000). Transcriptional dysregulation in Huntington’s disease. Trends Neurosci. 23, 387–392 10.1016/s0166-2236(00)01609-x 10941183

[B7] Chen Y. T. Zang X. F. Pan J. Zhu X. L. Chen F. Chen Z. B. (2012). Expression patterns of histone deacetylases in experimental stroke and potential targets for neuroprotection. Clin. Exp. Pharmacol. Physiol. 39, 751–758 10.1111/j.1440-1681.2012.05729.x 22651689

[B8] Corcoran L. J. Mitchison T. J. Liu Q. (2004). A novel action of histone deacetylase inhibitors in a protein aggresome disease model. Curr. Biol. 14, 488–492 10.1016/j.cub.2004.03.003 15043813

[B9] Corona J. C. Tovar-y-Romo L. B. Tapia R. (2007). Glutamate excitotoxicity and therapeutic targets for amyotrophic lateral sclerosis. Expert Opin. Ther. Targets 11, 1415–14128 10.1517/14728222.11.11.1415 18028007

[B10] Cudkowicz M. E. Andres P. L. Macdonald S. A. Bedlack R. S. Choudry R. Brown R. H. (2009). Phase 2 study of sodium phenylbutyrate in ALS. Amyotroph. Lateral Scler. 10, 99–106 10.1080/17482960802320487 18688762

[B11] Dasgupta B. Milbrandt J. (2007). Resveratrol stimulates AMP kinase activity in neurons. Proc. Natl. Acad. Sci. U S A 104, 7217–7222 10.1073/pnas.0610068104 17438283 PMC1855377

[B12] de Ruijter A. J. van Gennip A. H. Caron H. N. Kemp S. van Kuilenburg A. B. (2003). Histone deacetylases (HDACs): characterization of the classical HDAC family. Biochem. J. 370, 737–749 10.1042/bj20021321 12429021 PMC1223209

[B13] Del Signore S. J. Amante D. J. Kim J. Stack E. C. Goodrich S. Cormier K. (2009). Combined riluzole and sodium phenylbutyrate therapy in transgenic amyotrophic lateral sclerosis mice. Amyotroph. Lateral Scler. 10, 85–94 10.1080/17482960802226148 18618304

[B14] Eijkelenboom A. Burgering B. M. (2013). FOXOs: signalling integrators for homeostasis maintenance. Nat. Rev. Mol. Cell Biol. 14, 83–97 10.1038/nrm3507 23325358

[B15] Figueroa-Romero C. Hur J. Bender D. E. Delaney C. E. Cataldo M. D. Smith A. L. (2012). Identification of epigenetically altered genes in sporadic amyotrophic lateral sclerosis. PLoS One 7:e52672 10.1371/journal.pone.0052672 23300739 PMC3530456

[B16] Frew A. J. Johnstone R. W. Bolden J. E. (2009). Enhancing the apoptotic and therapeutic effects of HDAC inhibitors. Cancer Lett. 280, 125–133 10.1016/j.canlet.2009.02.042 19359091

[B17] Haigis M. C. Sinclair D. A. (2010). Mammalian sirtuins: biological insights and disease relevance. Annu. Rev. Pathol. 5, 253–295 10.1146/annurev.pathol.4.110807.092250 20078221 PMC2866163

[B18] Hubbert C. Guardiola A. Shao R. Kawaguchi Y. Ito A. Nixon A. (2002). HDAC6 is a microtubule-associated deacetylase. Nature 417, 455–458 10.1038/417455a 12024216

[B19] Imai S. Armstrong C. M. Kaeberlein M. Guarente L. (2000). Transcriptional silencing and longevity protein Sir2 is an NAD-dependent histone deacetylase. Nature 403, 795–800 10.1038/35001622 10693811

[B20] Janssen C. Schmalbach S. Boeselt S. Sarlette A. Dengler R. Petri S. (2010). Differential histone deacetylase mRNA expression patterns in amyotrophic lateral sclerosis. J. Neuropathol. Exp. Neurol. 69, 573–581 10.1097/nen.0b013e3181ddd404 20467334

[B21] Jiang M. Wang J. Fu J. Du L. Jeong H. West T. (2012). Neuroprotective role of Sirt1 in mammalian models of Huntington’s disease through activation of multiple Sirt1 targets. Nat. Med. 18, 153–158 10.1038/nm.2558 22179319 PMC4551453

[B22] Kaeberlein M. McVey M. Guarente L. (1999). The SIR2/3/4 complex and SIR2 alone promote longevity in Saccharomyces cerevisiae by two different mechanisms. Genes Dev. 13, 2570–2580 10.1101/gad.13.19.2570 10521401 PMC317077

[B23] Kanai H. Sawa A. Chen R. W. Leeds P. Chuang D. M. (2004). Valproic acid inhibits histone deacetylase activity and suppresses excitotoxicity-induced GAPDH nuclear accumulation and apoptotic death in neurons. Pharmacogenomics J. 4, 336–344 10.1038/sj.tpj.6500269 15289798

[B24] Kazantsev A. G. Thompson L. M. (2008). Therapeutic application of histone deacetylase inhibitors for central nervous system disorders. Nat. Rev. Drug Discov. 7, 854–868 10.1038/nrd2681 18827828

[B25] Kernochan L. E. Russo M. L. Woodling N. S. Huynh T. N. Avila A. M. Fischbeck K. H. (2005). The role of histone acetylation in SMN gene expression. Hum. Mol. Genet. 14, 1171–1182 10.1093/hmg/ddi130 15772088

[B26] Kim D. Nguyen M. D. Dobbin M. M. Fischer A. Sananbenesi F. Rodgers J. T. (2007). SIRT1 deacetylase protects against neurodegeneration in models for Alzheimer’s disease and amyotrophic lateral sclerosis. EMBO J. 26, 3169–3179 10.1038/sj.emboj.7601758 17581637 PMC1914106

[B27] Kim S. H. Shanware N. P. Bowler M. J. Tibbetts R. S. (2010). Amyotrophic lateral sclerosis-associated proteins TDP-43 and FUS/TLS function in a common biochemical complex to co-regulate HDAC6 mRNA. J. Biol. Chem. 285, 34097–34105 10.1074/jbc.m110.154831 20720006 PMC2962508

[B28] Kirby J. Halligan E. Baptista M. J. Allen S. Heath P. R. Holden H. (2005). Mutant SOD1 alters the motor neuronal transcriptome: implications for familial ALS. Brain 128, 1686–1706 10.1093/brain/awh503 15872021

[B29] Kouzarides T. (2007). Chromatin modifications and their function. Cell 128, 693–705 10.1016/j.cell.2007.02.005 17320507

[B30] Lee J. C. Shin J. H. Park B. W. Kim G. S. Kim J. C. Kang K. S. (2012). Region-specific changes in the immunoreactivity of SIRT1 expression in the central nervous system of SOD1(G93A) transgenic mice as an in vivo model of amyotrophic lateral sclerosis. Brain Res. 1433, 20–28 10.1016/j.brainres.2011.11.019 22137654

[B31] Li X. (2013). SIRT1 and energy metabolism. Acta Biochim. Biophys. Sin. (Shanghai) 45, 51–60 10.1093/abbs/gms108 23257294 PMC3527007

[B32] Liang H. Ward W. F. Jang Y. C. Bhattacharya A. Bokov A. F. Li Y. (2011). PGC-1alpha protects neurons and alters disease progression in an amyotrophic lateral sclerosis mouse model. Muscle Nerve 44, 947–956 10.1002/mus.22217 22102466

[B33] Liu D. Gharavi R. Pitta M. Gleichmann M. Mattson M. P. (2009a). Nicotinamide prevents NAD^+^ depletion and protects neurons against excitotoxicity and cerebral ischemia: NAD^+^ consumption by SIRT1 may endanger energetically compromised neurons. Neuromolecular Med. 11, 28–42 10.1007/s12017-009-8058-1 19288225 PMC2677622

[B34] Liu H. Hu Q. D’Ercole A J. Ye P. (2009b). Histone deacetylase 11 regulates oligodendrocyte-specific gene expression and cell development in OL-1 oligodendroglia cells. Glia 57, 1–12 10.1002/glia.20729 18627006 PMC2595137

[B35] Liu H. Hu Q. Kaufman A. D’Ercole A. J. Ye P. (2008). Developmental expression of histone deacetylase 11 in the murine brain. J. Neurosci. Res. 86, 537–543 10.1002/jnr.21521 17893925 PMC2254516

[B36] Michan S. Sinclair D. (2007). Sirtuins in mammals: insights into their biological function. Biochem. J. 404, 1–13 10.1042/bj20070140 17447894 PMC2753453

[B37] Mojsilovic-Petrovic J. Nedelsky N. Boccitto M. Mano I. Georgiades S. N. Zhou W. (2009). FOXO3a is broadly neuroprotective in vitro and in vivo against insults implicated in motor neuron diseases. J. Neurosci. 29, 8236–8247 10.1523/jneurosci.1805-09.2009 19553463 PMC2748231

[B38] Monti B. Polazzi E. Contestabile A. (2009). Biochemical, molecular and epigenetic mechanisms of valproic acid neuroprotection. Curr. Mol. Pharmacol. 2, 95–109 10.2174/1874-470210902010095 20021450

[B39] Pandey U. B. Nie Z. Batlevi Y. McCray B. A. Ritson G. P. Nedelsky N. B. (2007). HDAC6 rescues neurodegeneration and provides an essential link between autophagy and the UPS. Nature 447, 859–863 10.1038/nature05853 17568747

[B40] Petri S. Kiaei M. Kipiani K. Chen J. Calingasan N. Y. Crow J. P. (2006). Additive neuroprotective effects of a histone deacetylase inhibitor and a catalytic antioxidant in a transgenic mouse model of amyotrophic lateral sclerosis. Neurobiol. Dis. 22, 40–49 10.1016/j.nbd.2005.09.013 16289867

[B41] Piepers S. Veldink J. H. de Jong S. W. van der Tweel I. van der Pol W. L. Uijtendaal E. V. (2009). Randomized sequential trial of valproic acid in amyotrophic lateral sclerosis. Ann. Neurol. 66, 227–234 10.1002/ana.21620 19743466

[B42] Robakis N. K. (2003). An Alzheimer’s disease hypothesis based on transcriptional dysregulation. Amyloid 10, 80–85 10.3109/13506120309041729 12964415

[B43] Robberecht W. Philips T. (2013). The changing scene of amyotrophic lateral sclerosis. Nat. Rev. Neurosci. 14, 248–264 10.1038/nrn3430 23463272

[B44] Rouaux C. Panteleeva I. Rene F. Gonzalez de Aguilar J. L. Echaniz-Laguna A. Dupuis L. (2007). Sodium valproate exerts neuroprotective effects in vivo through CREB-binding protein-dependent mechanisms but does not improve survival in an amyotrophic lateral sclerosis mouse model. J. Neurosci. 27, 5535–5545 10.1523/jneurosci.1139-07.2007 17522299 PMC6672753

[B45] Ryu H. Smith K. Camelo S. I. Carreras I. Lee J. Iglesias A. H. (2005). Sodium phenylbutyrate prolongs survival and regulates expression of anti-apoptotic genes in transgenic amyotrophic lateral sclerosis mice. J. Neurochem. 93, 1087–1098 10.1111/j.1471-4159.2005.03077.x 15934930

[B46] Santa-Cruz L. D. Ramírez-Jarquín U. N. Tapia R. (2012). “Role of mitochondrial dysfunction in motor neuron degeneration in ALS,” in Amyotrophic Lateral Sclerosis, ed Maurer M. H. (In Tech, Rijeka, Croatia). 197–224

[B47] Selvi B. R. Cassel J. C. Kundu T. K. Boutillier A. L. (2010). Tuning acetylation levels with HAT activators: therapeutic strategy in neurodegenerative diseases. Biochim. Biophys. Acta 1799, 840–853 10.1016/j.bbagrm.2010.08.012 20833281

[B48] Smith J. S. Brachmann C. B. Celic I. Kenna M. A. Muhammad S. Starai V. J. (2000). A phylogenetically conserved NAD+-dependent protein deacetylase activity in the Sir2 protein family. Proc. Natl. Acad. Sci. U S A 97, 6658–6663 10.1073/pnas.97.12.6658 10841563 PMC18692

[B49] Song W. Song Y. Kincaid B. Bossy B. Bossy-Wetzel E. (2013). Mutant SOD1G93A triggers mitochondrial fragmentation in spinal cord motor neurons: neuroprotection by SIRT3 and PGC-1alpha. Neurobiol. Dis. 51, 72–81 10.1016/j.nbd.2012.07.004 22819776 PMC3992938

[B50] Tang B. L. (2010). Resveratrol is neuroprotective because it is not a direct activator of Sirt1- A hypothesis. Brain Res. Bull. 81, 359–361 10.1016/j.brainresbull.2009.12.007 20026255

[B51] Tanno M. Sakamoto J. Miura T. Shimamoto K. Horio Y. (2007). Nucleocytoplasmic shuttling of the NAD+-dependent histone deacetylase SIRT1. J. Biol. Chem. 282, 6823–6832 10.1074/jbc.m609554200 17197703

[B52] Thau N. Knippenberg S. Korner S. Rath K. J. Dengler R. Petri S. (2012). Decreased mRNA expression of PGC-1alpha and PGC-1alpha-regulated factors in the SOD1G93A ALS mouse model and in human sporadic ALS. J. Neuropathol. Exp. Neurol. 71, 1064–1074 10.1097/nen.0b013e318275df4b 23147503

[B53] Todd P. K. Oh S. Y. Krans A. Pandey U. B. Di Prospero N. A. Min K. T. (2010). Histone deacetylases suppress CGG repeat-induced neurodegeneration via transcriptional silencing in models of fragile X tremor ataxia syndrome. PLoS Genet. 6:e1001240 10.1371/journal.pgen.1001240 21170301 PMC3000359

[B54] Tovar-y-Romo L. B. Zepeda A. Tapia R. (2007). Vascular endothelial growth factor prevents paralysis and motoneuron death in a rat model of excitotoxic spinal cord neurodegeneration. J. Neuropathol. Exp. Neurol. 66, 913–922 10.1097/nen.0b013e3181567c16 17917585

[B55] Wang J. Zhang Y. Tang L. Zhang N. Fan D. (2011). Protective effects of resveratrol through the up-regulation of SIRT1 expression in the mutant hSOD1–G93A-bearing motor neuron-like cell culture model of amyotrophic lateral sclerosis. Neurosci. Lett. 503, 250–255 10.1016/j.neulet.2011.08.047 21896316

[B56] Yacoubian T. A. Cantuti-Castelvetri I. Bouzou B. Asteris G. McLean P. J. Hyman B. T. (2008). Transcriptional dysregulation in a transgenic model of Parkinson disease. Neurobiol. Dis. 29, 515–528 10.1016/j.nbd.2007.11.008 18191405 PMC2707844

[B57] Yao Z. G. Zhang L. Huang L. Zhu H. Liu Y. Ma C. M. (2013). Regional and cell-type specific distribution of HDAC2 in the adult mouse brain. Brain Struct. Funct. 218, 563–573 10.1007/s00429-012-0416-3 22532304

[B58] Yoo Y. E. Ko C. P. (2011). Treatment with trichostatin A initiated after disease onset delays disease progression and increases survival in a mouse model of amyotrophic lateral sclerosis. Exp. Neurol. 231, 147–159 10.1016/j.expneurol.2011.06.003 21712032

[B59] Zakhary S. M. Ayubcha D. Dileo J. N. Jose R. Leheste J. R. Horowitz J. M. (2010). Distribution analysis of deacetylase SIRT1 in rodent and human nervous systems. Anat. Rec. (Hoboken) 293, 1024–1032 10.1002/ar.21116 20225204 PMC3071026

[B60] Zhao W. Varghese M. Yemul S. Pan Y. Cheng A. Marano P. (2011a). Peroxisome proliferator activator receptor gamma coactivator-1alpha (PGC-1alpha) improves motor performance and survival in a mouse model of amyotrophic lateral sclerosis. Mol. Neurodegener. 6:51 10.1186/1750-1326-6-51 21771318 PMC3156746

[B61] Zhao Y. Wang Y. Zhu W. G. (2011b). Applications of post-translational modifications of FoxO family proteins in biological functions. J. Mol. Cell Biol. 3, 276–282 10.1093/jmcb/mjr013 21669942

[B62] Zhou V. W. Goren A. Bernstein B. E. (2011). Charting histone modifications and the functional organization of mammalian genomes. Nat. Rev. Genet. 12, 7–18 10.1038/nrg2905 21116306

